# Brief cognitive behavioral therapy compared to general practitioners care for depression in primary care: a randomized trial

**DOI:** 10.1186/1745-6215-11-96

**Published:** 2010-10-12

**Authors:** Kim D Baas, Maarten WJ Koeter, Henk C van Weert, Peter Lucassen, Claudi LH Bockting, Karin A Wittkampf, Aart H Schene

**Affiliations:** 1 Department of Psychiatry, Academic Medical Center, University of Amsterdam, Amsterdam, The Netherlands; 2 Department of General Practice, Academic Medical Centre, University of Amsterdam, Amsterdam, The Netherlands; 3 Department of Primary and Community Care, Radboud University Nijmegen Medical Centre, Nijmegen, The Netherlands; 4 Department of Clinical Psychology, Groningen University, Groningen, The Netherlands

## Abstract

**Background:**

Depressive disorders are highly prevalent in primary care (PC) and are associated with considerable functional impairment and increased health care use. Research has shown that many patients prefer psychological treatments to pharmacotherapy, however, it remains unclear which treatment is most optimal for depressive patients in primary care.

**Methods/Design:**

A randomized, multi-centre trial involving two intervention groups: one receiving brief cognitive behavioral therapy and the other receiving general practitioner care. General practitioners from 109 General Practices in Nijmegen and Amsterdam (The Netherlands) will be asked to include patients aged between 18-70 years presenting with depressive symptomatology, who do not receive an active treatment for their depressive complaints. Patients will be telephonically assessed with the Structured Clinical Interview for DSM-IV Axis I Disorders (SCID-I) to ascertain study eligibility. Eligible patients will be randomized to one of two treatment conditions: either 8 sessions of cognitive behavioral therapy by a first line psychologist or general practitioner's care according to The Dutch College of General Practitioners Practice Guideline (NHG- standaard). Baseline and follow-up assessments are scheduled at 0, 6, 12 and 52 weeks following the start of the intervention. Primary outcome will be measured with the Hamilton Depression Rating Scale-17 (HDRS-17) and the Patient Health Questionnaire-9 (PHQ-9). Outcomes will be analyzed on an intention to treat basis.

**Trial Registration:**

ISRCTN65811640

## Background

Depressive disorders are highly prevalent in primary care (PC) [1,2] and are associated with considerable impairment in quality of life [3,4], high service utilization use and high medical costs [5]. Most patients with depressive disorders are seen and treated by their general practitioner (GP) [6,7].

The Dutch multidisciplinary guideline (2005) for Major Depressive Disorder (MDD) and the Dutch College of General Practitioners Practice Guideline offer evidence based treatments for PC patients. Two options are available: brief psychotherapy and a minimal intervention by the GP, which may include prescribing of antidepressants. The latter will be referred to as general practitioners care (GPC).

Prescribing of antidepressants is dependent of suffering, dysfunction and the preference of patients. However, research has shown that nowadays many patients prefer psychological treatments to pharmacotherapy [8,9].

Effective psychological treatments, like brief cognitive behavioural therapy (bCBT), for depression are available in PC [10]. However, little is known about the relative effectiveness of a bCBT compared to GPC. This is most likely due to limitations in carrying out bCBT studies in PC. These limitations include for example specialized and standardized training of primary care professionals in the intervention.

In the Netherlands, all inhabitants are enlisted with a GP and have an open and unlimited access to the GP. The GP performs a gatekeeper function which enables him to control access to specialist health care. First line psychologists (FLP) are operating in PC and are often connected to the practices of the GP. They offer generalized psychological care, mostly after referral of a GP, and are easily accessible.

The few studies that are available concerning the benefits of bCBT relative to GPC for treating depression in primary care show conflicting results.

Proutfoot *et al*, Schoenbaum *et al *and Simon *et al *showed benefits of bCBT relative to GPC [11-13], while there is also some evidence showing that bCBT might not be superior to GPC at post treatment [14] and at 12-month follow-up [15].

## Methods/Design

### Objective

The aim of this paper is to present the research protocol of our randomized trial designed to compare a protocolized brief CBT (bCBT) performed by a FLP with a treatment performed by a general practitioner (general practitioners care; GPC) for primary care patients with MDD. We hypothesize that bCBT will be superior to GPC.

### Design and setting

Primary care patients with MDD will be randomized to either an eight session bCBT or GPC of 12 weeks duration.

The study will be conducted in 109 General Practices in two regions connected to academic settings in The Netherlands: the Academic Medical Center in Amsterdam and the Radboud University Nijmegen Medical Center in Nijmegen.

### Inclusion criteria

Patients aged between 18 and 70 years and suffering from MDD as determined by an independent researcher with the Structured Clinical Interview for DSM-IV Axis I Disorders (SCID-I) [16,17].

### Exclusion criteria

- Suffering from schizophrenia or bipolar disorder (now and in the past as assessed by the SCID-I).

- Contra-indication for MDD treatment like mental retardation or terminal illness.

- Insufficient comprehension of the Dutch or English language.

- Severe suicidal thoughts.

- Receiving an active MDD treatment. Active treatment is defined as:

- Antidepressive medication (except those who receive this in low dosage for pain complaints or as an anti-smoking intervention: Amitriptyline ≤ 50 mg, Nortriptyline ≤ 50 mg, Zyban (bupropion)).

- Psychotherapy.

- Supportive consultations by the GP or social worker, except for diagnostic visits to complete the diagnosis (≤ 2).

### Recruitment of patients and baseline assessment

GPs will be asked to include patients whom they consider to be suffering from MDD. Patients identified by their GP as suffering from MDD will receive information regarding the study and are asked to fill out the Patient Health Questionnaire (PHQ) [18]. Within 3 days after inclusion by the GP, a researcher will contact the patient by telephone to provide further explanation and seek informed consent regarding the diagnostic phase of the study. Consenting patients will be further assessed with a telephonic SCID-I interview. If the patient fulfills the DSM-IV criteria for MDD, subsequent SCID-I sections will be checked to assess eligibility. When eligible, the patient will be further assessed with a baseline Hamilton Depression Rating Scale-17 (HDRS-17) [19] to establish the severity of the depression. At the end of the telephonic assessment the patient receives the results of the interview and is asked to discuss these results and the treatment options with the GP. All results will also be sent to the GP. During the appointment with the GP the patient will make the decision to enter the trial or not. If the patient agrees to participate in the study, a second informed consent form will be signed and the GP proceeds with the randomisation procedure as described below.

### Randomization

After informed consent, GPs will perform an internet based randomization procedure. A computer program generates a stratified block randomization (block randomization of 4 blocks stratified by gender and location (Nijmegen/Amsterdam)) where the patient is randomized over the two treatment strategies with no preference option. The computer then generates an outcome on the GP's computer screen and simultaneously sends an email of this outcome to the researcher. If the patient is randomized to GPC, the GP will immediately make the first appointment. If the patient is randomized to bCBT the researcher will contact the nearest participating FLP and refer the patient.

### Interventions

#### General Practitioners Care

The treatment protocol is set up in accordance with the Dutch College of General Practitioners Practice Guideline (NHG- standaard) [20] and composed after evaluating the content of colleague general practitioners' (follow up) appointments with depressive patients.

The description of the content of the contacts is meant as a guideline; GP's can adapt the scheme to their own style or to special circumstances. The treatment consists of supportive contacts which can be combined with an antidepressant agent. The duration of the treatment is 12 weeks. The following elements are recommended: psycho-education about depression, life style advices about sleep, alcohol/drugs, nutrition, social activities and physical activities.

The minimum frequency is one contact every two weeks during the first six weeks and one telephonic contact and one face- to- face evaluation contact during the second 6 week period. This can be increased if needed. Reasons for more contacts can be for instance: the severity of the complaints and/or lack of social support. After the first six weeks the GP evaluates, together with the patient, the need and frequency for further contacts. If recovery is not sufficient, according to the patient and/or GP, the GP will offer further contacts, during which problem solving will be a key element. If recovery is substantial the GP will provide information on relapse prevention and will offer contacts by telephone during the next month and one face-to- face contact after six weeks.

See table 1 for an overview of the contents of the GPC.

**Table 1:** Overview of the contents of the GPC

**Session nr**.	Required elements
Contact 1	Discuss the diagnosis, feelings of the patient and psycho-education (with or without an antidepressant agent)
Contact 2	Evaluation, further psycho-education and life style advices.
Contact 3	Evaluation, re-enforcement and follow-up appointments.
	Thereafter if recovery is:
Contact 4+5	Sufficient: contact by telephone to discuss the course and evaluate possible relapse. Insufficient: evaluation, reinforcement and problem solving
Contact 6	Sufficient: evaluation, relapse prevention Insufficient: evaluation, relapse prevention/offer pharmacological treatment

To monitor the actual content of the provided GPC and potential supplementary treatment, we will sent each GP a short questionnaire at the end of the 12 weeks in which they can state how many appointments they have had with the patient, whether they prescribed an antidepressant (and the dosage) and whether they combined their treatment with a treatment by another health care professional or referred their patient.

In case of randomisation to the FLP, GPs will be asked to fill in a short questionnaire at the end of the bCBT in which they can state whether they combined the bCBT with an antidepressant (and the dosage) and whether they combined the bCBT treatment with a treatment by another health care professional or referred the patient.

### Training

In order to optimise the treatment we will pay each participating GP a visit before the start of the study. During this visit we will explain the treatment protocol, content of the contacts and provide a ring binder with the protocol. They will also be informed about the possibility of consulting an independent physician for questions during the intervention period.

GPs will be instructed to make a call to the researcher if they want to divert from the protocol (for example referring a patient to secondary care or a social worker).

### Brief Cognitive Behavioural Therapy (bCBT)

The treatment will consist of 8 sessions within 12 weeks, each of fifty minutes duration. At the end of each session patients will receive homework assignments. The treatment will be directed at the role of behaviour and thinking in depressive complaints. Behaviour: patients will obtain insight in the role of pleasant activities on mood and subsequently learn to identify and expand potentially pleasant activities. Thinking: patients will obtain insight in the influence of negative thoughts and beliefs on their feelings/mood and subsequently learn to challenge these thoughts and beliefs thereby reducing the impact on their feelings. The patient learns to formulate alternative (rational) thoughts and beliefs. Eventually, the therapy will result in a personal prevention plan.

All therapists are licensed first line psychologists trained in this form of cognitive behavioural therapy. To guarantee quality, the sessions will be audio taped and the integrity of the intervention will be assessed. Also, the therapists will perform the intervention under supervision, which means that the therapists will regularly discuss the sessions with colleagues.

See table 2 for an overview of the contents of the bCBT.

**Table 2:** Overview of the contents of the bCBT

**Session nr**.	Required elements
Session 1	- Psycho- education - Explanation of the relation between pleasant activities and mood - Explanation of registration activities - Homework
Session 2	- Review homework assignment - Identification and expanding of potentially pleasant activities. - Explanation of the relation between cognitions and feelings. - Explanation 3 columns - Homework
Session 3	- Review homework assignment - Explain challenging negative thoughts/formulating rational thoughts - Explanation 5 columns - Homework
Session 4	- Review homework assignment - Explain challenge tactics again. - Explanation 7 columns - Homework
Session 5	- Review homework assignment - Practice challenging negative thoughts/formulating rational thoughts - Optional: explanation of schema's - Homework
Session 6	- Review homework assignment - Explanation and challenging of schema's - Homework
Session 7	- Review homework assignment - Prevention plan - Homework
Session 8	- Review homework assignment - How further and prevention plan. - Evaluation and farewell.

### Training

The FLPs will be trained in the treatment protocol by an experienced cognitive behavioural therapist during a two day course. After this course a booster session is planned (by then all therapist have performed at least one 8- session treatment) where problems and questions encountered during the treatment will be addressed.

### Assessments

Patients will be assessed on four occasions: at study entry (t0), 6 weeks after the start of the intervention (t1), at the end of the intervention period (i.e. 12 weeks after the start of the intervention (t2)) and at 52 weeks after the start of the intervention (follow-up assessment;t3).

Assessments will be performed by experienced research interviewers who are blind for the type of treatment given. If a patient drops out of treatment an exit assessment with the outcome measure (see paragraph primary outcome measures) will be completed if possible. The following socio- demographic data will be collected: sex, age, ethnicity, marital status, education, primary role and living situation.

### Eligibility assessment

The *Structured Clinical Interview for DSM-IV Axis I disorders *(SCID-I) is a semi-structured interview designed for diagnosing mental disorders according to DSM-IV criteria. Agreement between diagnoses based on telephone and face to face administration of the SCID-I is good (Kappa = 0.73 (with 90% agreement) [21]. The SCID-I will be administered by researchers that have received a SCID-I training from a professional that received an official SCID training. Throughout the study all interviewers will participate in ongoing training sessions and in monthly consensus meetings to maximize accuracy and consistency in the administration of the SCID-I.

### Primary outcome measures

Primary outcomes will be depressive symptomatology as measured by the patient rated PHQ-9 [22], depression severity as measured by the clinician rated HDRS-17 [19], the proportion of patients achieving response on the HDRS-17 and the proportion of patients achieving remission on the HDRS-17. In accordance with the National Institute of Mental Health criteria [23], response is defined as a ≥ 50% decrease of the baseline HDRS-17 score, remission is defined as a HDRS-17 score ≤7.

#### *Hamilton Depression Rating Scale- 17 *(HDRS-17)

The HDRS-17 is a widely used semi- structured clinical interview covering a range of affective, behavioral and biological symptoms and has acceptable psychometric properties [19]. Throughout the study all HDRS-17 interviewers will participate in ongoing training sessions and monthly consensus meetings supervised by an expert psychiatrist to maximize accuracy and consistency in the administration of the HDRS-17.

#### *The Patient Health Questionnaire-9 *(PHQ-9)

The PHQ is a relatively short self report version of the Primary Care Evaluation of Mental Disorders (PRIME-MD). The full PHQ screens for the five most common mental disorders using DSM-IV criteria: depressive disorders (MDD and dysthymia), anxiety disorders (panic disorder and generalized anxiety disorder), alcohol abuse, somatoform disorder, and eating disorders (bulimia nervosa and binge eating disorder) [18]. The PHQ-9 is the 9 item sub-scale for MDD [22].

### Secondary outcome measures

The secondary outcome measure is quality of life. It will be measured with the MOS-SF36, a short-form health survey consisting of 36 questions. It yields an 8-scale profile of functional health and well-being as well as a physical health and mental health summary scale and a preference-based health utility index. The MOS-SF-36 has been successfully used in surveys of general and specific populations, to compare the relative burden of diseases, and to assess and compare the health benefits produced by a wide range of different treatments [24].

### Power

Based on Scott *et al *[25] we expect a medium effect size (.50) [26] of bCBT as compared to GPC. With an alpha of 0.05 (one-sided) we will need 51 patients in each experimental condition to achieve a power of .80 [27].

When taking into account a refusal rate of .20 [25,28-30] we will need 61 patients per treatment condition, which implies a total sample size of 122 patients.

### Data analysis

We will compare the groups at baseline on socio- demographic characteristics, and functional and clinical data to check whether the randomization has been successful. The main analyses will be intention to treat.

### Treatment effect

To take potential biased outcomes caused by selective loss to follow-up into account we will use a generalized linear mixed model (GLMM) analysis approach, which, assuming missing at random (MAR) for missing values, gives unbiased effect estimates. MAR is a less restrictive assumption than missing completely at random and allows loss to follow-up to be related to baseline characteristics that are incorporated in the regression model. In all of these analyses intervention is the independent variable and a propensity score is entered as covariate to adjust for potential confounders. All analyses with dichotomous outcomes will be carried out with Generalized Estimating Equations (GEE).

### Ethical aspects

The study protocol is approved by the institutional ethics review committee of both participating centers.

Informed consent will be obtained from the participants at two moments in the study, namely before entering the study and before randomization. Prior to giving consent, the patients will be provided with all the aims and characteristics of the study and the two interventions.

For the patients randomized to the bCBT there will be another informed consent moment. Namely, before the start of the therapy informed consent will be obtained for audio taping the sessions.

Finally, all patients will be informed that participation is voluntary and that they are able to withdraw at any time.

## Competing interests

The authors declare that they have no competing interests.

## Authors' contributions

All authors were responsible for the development of the study design. AS was responsible for the funding. MK and HvW are the trial coordinators responsible for the ongoing management of the trial. KB, HvW, PL and CB were responsible for the development of the intervention and the training of the first line psychologists and general practitioners. MK and KB developed the statistical analyses. KB wrote the initial draft manuscript. All authors have read and corrected the draft versions and all authors contributed to and approved the final manuscript.
